# Does Bariatric Surgery Normalize Risks After Total Knee Arthroplasty? Administrative Medicare Data

**DOI:** 10.5435/JAAOSGlobal-D-19-00102

**Published:** 2019-12-24

**Authors:** Menachem M. Meller, Stuart Goodman, Mark H. Gonzalez, Edmund Lau

**Affiliations:** From the Main Line Health, Section of orthopedic surgery, Merion Station, PA (Dr. Meller); the Stanford University Medical Center, Department of Orthopedic Surgery and Bioengineering, Redwood City, CA (Dr. Goodman), the University of Illinois, Department of Orthopedic Surgery, Chicago, Il,(Dr. Gonzalez), Exponent Inc, Health Sciences, Menlo Park, Ca (Mr. Lau).

## Abstract

Patients with morbid obesity, defined as body mass index of greater than 40 kg/m^2^, are being referred for weight loss and bariatric surgery before being accepted for a total knee arthroplasty (TKA). Previous studies have identified the risks associated with doing a TKA in an individual with an increased body mass index. We now present data identifying the same risks in individuals who have undergone bariatric surgery before submitting to TKA.

**Questions/Purposes:**

(1) Has the bariatric surgery improved the risk profile for the subsequent TKA? (2) Does the type of bariatric procedure matter?

**Method:**

A retrospective cohort study was conducted of patients who underwent bariatric surgery followed by TKA using Medicare hospital claims data. A study was undertaken using the Current Procedure Terminology codes and *International Classification of Diseases-9* and *International Classification of Diseases-10* for bariatric surgery. These identified entries were then cross-referenced to individuals who later underwent TKA, identified by CPT 27447, between 2004 and 2016. Twelve different types of complications which occurred in the 90-day period after the TKA were analyzed.

**Results:**

Postbariatric bypass surgery patients showed a markedly elevated risk in most complications examined. In each category, the type of previous gastric surgery had notable differences in the post-TKA complication profile. In the implant failure category, the data demonstrated an even greater risk after a gastric bypass. When postbariatric patients were compared with morbidly obese individuals who had not undergone bariatric surgery, the hazard ratios (HRs) were markedly elevated for death (HR 1.47/bypass), implant failure (HR 1.58/sleeve), and pneumonia (HR 1.68/bypass).

**Conclusion:**

(1) Submitting to bariatric surgery is not sufficient to normalize risks. (2) The type of previous bariatric procedure is associated with the type of complications encountered. (3) We were unable to attribute TKA to bariatric failures. (4) Health systems and health care providers should be cautious in withholding care for patients with morbid obesity.

Total knee arthroplasty (TKA) is a common treatment for end-stage degenerative joint disease. Obesity has been demonstrated to increase the costs and complications, including death, associated with the performance of a TKA.^1^

Efforts to mitigate risks include programs to optimize outcomes by addressing so-called modifiable risk factors.^2^ Obesity, defined as body mass index (BMI) of > 30 kg/m^2^, is a possible modifiable risk factor. Efforts to modify the level of obesity have included recommendations for submitting to bariatric surgery before undergoing a TKA. The types of bariatric surgical procedures being done include open or laparoscopic banding, sleeve, and Roux-en-Y bypasses.

In this study, we hypothesized that postbariatric surgical patients have persistently elevated complications when compared with routine TKA. We have also hypothesized that the more aggressive gastric bypass procedures and persistently elevated BMI correspond to even higher postoperative costs and complications. In this study, when the general term “gastric bypass” is being used, it refers to any gastric bypass, whereas the term “bypass” refers to a Roux-en-Y procedure (creating a small pouch from the stomach and connecting the newly created pouch directly to the small intestine) specifically.

## Methods

For this study, the Medicare inpatient claims data were used to identify patients who first underwent bariatric surgery followed by a TKA between January 1, 2004, and December 31, 2016. These patients were identified using the Current Procedure Terminology codes (CPT) and *International Classification of Diseases-9* (*ICD-9*) and *ICD-10*. These identified entries were then cross-referenced to individuals who later underwent TKA, identified by CPT 27447, between 2004 and 2016. Twelve different types of complications which occurred in the 90-day period after the TKA were analyzed. We have previously published a validation study^3^ and elements of the methods used.^4^

Patients who underwent previous bariatric surgery were identified by CPT 43770 (laparoscopic band), 43775 (laparoscopic sleeve), and 43664 (laparoscopic gastroenterostomy) and *ICD-9* Z98.84 and *ICD-10* V45.86. The laparoscopic procedure codes are ordered from mild to aggressive levels of gastric bypass. The codes Z-98.84 and V45.86 represented gastric bypasses where the type of bypass was not specified. These contain cases in which the bypass was done early in an open manner. In the absence of bypass diagnosis code, it was assumed that no bypass occurred.

There were two iterations used in this study design; the first compared the outcomes for postbariatric TKA with the entire TKA cohort, and the second compared the postbariatric TKA with a similar cohort with a BMI of greater than 40 kg/m^2^ who submitted to a TKA without previous bariatric surgery.

Twelve different complications that occurred during the 90-day postoperative period were examined. These output variables were selected to represent guidelines being used in process improvement, hospital, and physician performance. These variables have been previously reported on obesity outcome data.^4^ The scale and presentation was maintained to allow side-by-side comparisons of these risks with and without the bariatric surgery.

## Results

### Patient Characteristics

The Medicare LDS hospital data identified 2,701,427 primary knee arthroplasty records from 2004 to 2016 (Table 1). Of these, there were 25,852 patients (0.96%) who underwent a previous gastric bypass. The total annual number of pre-TKA gastric bypasses increased progressively from 86 in 2005 to 4,959 in 2016. The predominant bypass type was “other” 4,215 (1.72%), followed by sleeve 292 (0.12%), band 250 (0.10%), and gastric bypass 202 (0.08%). Table 2 summarizes the gastric bypass types and frequency of utilization of the cohort studied.

**Table 1:** Medicare Patients With TKA, 2005 to 2016, Demographic Profile and Previous Receipt of Bariatric Procedure

Effect	Level	Patients	Bariatric	% Bariatric	Avg. Year^a^
	Total	2,701,427	25,852	0.96	1.0
Age	65-69	807,860	15,696	1.94	0.7
	70-74	784,894	7,463	0.95	1.3
	75-79	626,535	2,092	0.33	1.8
	80+	482,138	601	0.12	2.4
Charlson Index	00	1,655,303	14,382	0.87	1.0
	1-2	883,650	9,108	1.03	1.0
	3-4	138,131	1,882	1.36	1.0
	5+	24,343	480	1.97	0.9
Hospital beds	000-149	774,952	7,640	0.99	1.1
	150-249	561,513	5,363	0.96	1.0
	250-449	806,176	7,582	0.94	1.0
	450+	558,786	5,267	0.94	1.0
Hospital ownership	Nonprofit	482,869	4,862	1.01	1.1
	Private	1,919,355	18,084	0.94	0.9
	Public	299,203	2,906	0.97	1.1
Hospital setting	Rural	340,586	2,807	0.82	1.2
	Urban	2,360,841	23,045	0.98	1.0
Hospital stay	1-2	563,624	6,564	1.16	1.0
	3-4	1,854,537	17,409	0.94	1.0
	5+	283,266	1,879	0.66	1.0
Race	Black	130,560	1,535	1.18	1.1
	Oth/unk	101,110	473	0.47	0.9
	White	2,469,757	23,844	0.97	1.0
Resident region	Midwest	765,842	6,714	0.88	1.0
	North East	422,300	3,706	0.88	0.9
	South	1,041,325	10,193	0.98	1.1
	West	471,960	5,239	1.11	0.9
Sex	Female	1,702,202	20,608	1.21	1.0
	Male	999,225	5,244	0.52	1.0
Year	2005	228,780	86	0.04	0.8
	2006	225,663	192	0.09	1.0
	2007	227,345	625	0.27	0.4
	2008	222,121	917	0.41	0.7
	2009	219,604	1,279	0.58	0.8
	2010	227,602	1,718	0.75	1.1
	2011	215,397	2,238	1.04	0.8
	2012	217,304	2,708	1.25	0.9
	2013	225,177	3,317	1.47	1.0
	2014	220,935	3,669	1.66	1.0
	2015	227,095	4,144	1.82	1.1
	2016	244,404	4,959	2.03	1.2

a Average time (year) from bariatric procedure to TKA.

**Table 2:** Types and Frequency of Bariatric Procedure Used Before TKA

	Band Gastroplasty		Gastric Bypass		Bariatric Procedure, Not Specified		Sleeve Gastrectomy		No Previous Bariatric Procedure	
	N	%	N	%	N	%	N	%	N	%
Total	2,044	0.08	1,671	0.06	21,112	0.78	1,025	0.04	2,675,575	99.04
2005	<10	0.00	72	0.03	<10	0.00	<10	0.00	228,694	99.96
2006	24	0.01	105	0.05	48	0.02	15	0.01	225,471	99.91
2007	49	0.02	86	0.04	464	0.20	26	0.01	226,720	99.73
2008	91	0.04	112	0.05	672	0.30	42	0.02	221,204	99.59
2009	178	0.08	132	0.06	918	0.42	51	0.02	218,325	99.42
2010	260	0.11	173	0.08	1,230	0.54	55	0.02	225,884	99.25
2011	230	0.11	133	0.06	1,818	0.84	57	0.03	213,159	98.96
2012	255	0.12	149	0.07	2,247	1.03	57	0.03	214,596	98.75
2013	241	0.11	148	0.07	2,842	1.26	86	0.04	221,860	98.53
2014	214	0.10	199	0.09	3,120	1.41	136	0.06	217,266	98.34
2015	245	0.11	160	0.07	3,538	1.56	201	0.09	222,951	98.18
2016	250	0.10	202	0.08	4,215	1.72	292	0.12	239,445	97.97
65-69	1,042	0.13	952	0.12	13,231	1.64	471	0.06	792,164	98.06
70-74	779	0.10	473	0.06	5,940	0.76	271	0.03	777,431	99.05
75-79	187	0.03	167	0.03	1,581	0.25	157	0.03	624,443	99.67
80+	36	0.01	79	0.02	360	0.07	126	0.03	481,537	99.88
Male	472	0.05	380	0.04	4,079	0.41	313	0.03	993,981	99.48
Female	1,572	0.09	1,291	0.08	17,033	1.00	712	0.04	1,681,594	98.79
Oth/unk	35	0.03	43	0.04	369	0.36	26	0.03	100,637	99.53
White	1,896	0.08	1,523	0.06	19,504	0.79	921	0.04	2,445,913	99.03
Black	113	0.09	105	0.08	1,239	0.95	78	0.06	129,025	98.82
Midwest	416	0.05	489	0.06	5,555	0.73	254	0.03	759,128	99.12
North east	291	0.07	187	0.04	3,077	0.73	151	0.04	418,594	99.12
South	972	0.09	686	0.07	8,076	0.78	459	0.04	1,031,132	99.02
West	365	0.08	309	0.07	4,404	0.93	161	0.03	466,721	98.89

### Ninety-Day Complications

**Figure 1:**
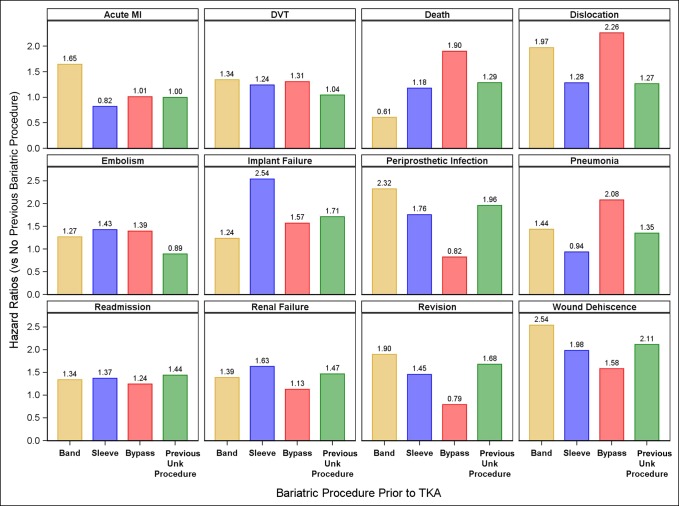
Complication rates expressed as hazard ratios, for 90 days after a TKA for each of the previous gastric bypass types, compared with all patients in the data set who have not undergone previous gastric bypass.

Patients who had undergone bariatric surgery were at increased risk of complications after a subsequent TKA when compared with the entire TKA cohort. Complications with a hazard ratio (HR) greater than 2.0 include dislocation, implant failure, periprosthetic infection, pneumonia, and wound dehiscence, Table 3 and Figure 1.

**Table 3:** HRs Comparing Use of Bariatric Procedures and Its Association With 90-Day Post-TKA Complications in Elderly Medicare Patients, 2004 to 2016

Complication	Bariatric Procedure (Reference: None)	HR	95% CI	*P* Value
Acute MI	Band gastroplasty	1.65	0.79-3.44	0.184
	Sleeve gastrectomy	0.82	0.21-3.28	0.780
	Gastric bypass	1.01	0.38-2.69	0.984
	Procedure not specified	1.00	0.73-1.73	0.985
DVT	Band gastroplasty	1.34	0.77-2.35	0.302
	Sleeve gastrectomy	1.24	0.56-2.76	0.596
	Gastric bypass	1.31	0.70-2.42	0.398
	Procedure not specified	1.04	0.85-1.29	0.685
Death	Band gastroplasty	0.61	0.20-1.88	0.385
	Sleeve gastrectomy	1.18	0.44-3.16	0.743
	Gastric bypass	1.90	1.00-3.64	0.052
	Procedure not specified	1.29	1.00-1.65	0.048
Dislocation	Band gastroplasty	1.97	0.68-6.13	0.241
	Sleeve gastrectomy	1.28	0.18-9.11	0.803
	Gastric bypass	2.26	0.73-7.03	0.158
	Procedure not specified	1.27	0.81-1.99	0.304
Embolism	Band gastroplasty	1.27	0.68-2.35	0.453
	Sleeve gastrectomy	1.43	0.64-3.18	0.386
	Gastric bypass	1.39	0.68-2.86	0.365
	Procedure not specified	0.89	0.70-1.13	0.345
Implant failure	Band gastroplasty	1.24	0.70-2.18	0.464
	Sleeve gastrectomy	2.54	1.45-4.46	0.001
	Gastric bypass	1.57	0.88-2.81	0.130
	Procedure not specified	1.71	1.47-1.99	<0.001
Periprosthetic infection	Band gastroplasty	2.32	1.54-3.50	<0.001
	Sleeve gastrectomy	1.76	0.92-3.37	0.090
	Gastric bypass	0.82	0.39-1.72	0.608
	Procedure not specified	1.96	1.69-2.26	<0.001
Pneumonia	Band gastroplasty	1.44	0.87-2.37	0.158
	Sleeve gastrectomy	0.94	0.42-2.09	0.871
	Gastric bypass	2.08	1.31-3.29	0.002
	Procedure not specified	1.35	1.14-1.59	<0.001
All cause readmission	Band gastroplasty	1.34	1.16-1.55	<0.001
	Sleeve gastrectomy	1.37	1.12-1.68	0.002
	Gastric bypass	1.24	1.06-1.46	0.007
	Procedure not specified	1.44	1.38-1.51	<0.001
Renal failure	Band gastroplasty	1.39	0.96-2.01	0.083
	Sleeve gastrectomy	1.63	1.06-2.51	0.026
	Gastric bypass	1.13	0.72-1.77	0.602
	Procedure not specified	1.47	1.31-1.64	<0.001
Revision	Band gastroplasty	1.90	1.22-2.94	0.004
	Sleeve gastrectomy	1.45	0.73-2.89	0.285
	Gastric bypass	0.79	0.38-1.66	0.539
	Procedure not specified	1.68	1.44-1.96	<0.001
Wound dehiscence	Band gastroplasty	2.54	1.59-4.05	<0.001
	Sleeve gastrectomy	1.98	0.95-4.13	0.068
	Gastric bypass	1.58	0.82-3.04	0.168
	Procedure not specified	2.11	1.78-2.52	<0.001

CI = confidence interval, HR = hazard ratio.

For death, the HR was elevated for the bypass, sleeve, and unknown categories and decreased for the band category (HR 1.90, 95% confidence interval [CI], 1.00 to 3.64; *P* < 0.052), (HR 1.18; 95% CI, 0.44 to 3.16, *P* < 0.743), (HR 1.29; 95% CI, 1.00 to 1.65, *P* < 0.048), and (HR 0.61; 95% CI, 0.20 to 1.88; *P* < 0.385), respectively. These HR corresponded to nine previous bypasses (of 1,671), four previous sleeve (of 1,025), 62 unknown (of 21,112), and three previous band procedures (out of 2,044).

The 90-day hospital readmission rate was elevated for all previous gastric bypass types including gastric banding (HR 1.34; 95% CI, 1.16 to 1.55; *P* < 0.001), gastric sleeve (HR 1.37; 95% CI, 1.12 to 1.68; *P* < 0.002), gastric bypass (Roux-en-Y) (HR 1.24; 95% CI, 1.06 to 1.46; *P* < 0.007), and previous type unknown (HR 1.44; 95% CI, 1.38 to 1.51; *P* < 0.001).

Table 4 presents the 90-day complication rates after TKA in patients who have undergone previous bariatric surgery. For all the 12 complications examined, patients who had undergone a previous gastric bypass type of procedure were at markedly elevated risk compared with having undergone no previous bypass. Complications that were not markedly elevated in postbariatric surgery patients were acute myocardial infarction after a sleeve, bypass, and “previous unknown,” death after a sleeve, embolism after “previous unknown,” periprosthetic infection after a bypass, and revision after a bypass. The incidence of an MI was 0 in the sleeve group, 1 of 380 in the bypass group, and 13 of 4,079 in the “other” group. Similar considerations were for the other low-risk categories.

**Table 4:** Use of Bariatric Procedure and Complications in 90-Day Post-TKA Complications in Elderly Medicare Patients, 2004 to 2016

Bariatric Procedure	TKA Patients	Death	DVT	Infection	Revision	Embolism	Implant Failure	Dehiscence	Acute MI	Renal Failure	Pneumonia	Dislocation	Readmission
None	2,675,575	9,395 (0.35%)	13,717 (0.51%)	12,094 (0.45%)	12,397 (0.46%)	10,669 (0.40%)	12,427 (0.46%)	7,417 (0.28%)	7,011 (0.26%)	24,356 (0.91%)	15,477 (0.58%)	1,776 (0.07%)	202,727 (7.58%)
Band gastroplasty	2,044	^a^	12 (0.59%)	23 (1.13%)	20 (0.98%)	10 (0.49%)	12 (0.59%)	17 (0.83%)	^a^	28 (1.37%)	15 (0.73%)	^a^	192 (9.39%)
Gastric bypass	1,671	^a^	10 (0.60%)	^a^	^a^	^a^	13 (0.78%)	^a^	^a^	19 (1.14%)	20 (1.20%)	^a^	156 (9.34%)
Other	21,112	62 (0.29%)	91 (0.43%)	192 (0.91%)	185 (0.88%)	72 (0.34%)	168 (0.80%)	148 (0.70%)	38 (0.18%)	312 (1.48%)	143 (0.68%)	21 (0.10%)	2,019 (9.56%)
Sleeve gastrectomy	1,025	^a^	^a^	^a^	^a^	^a^	12 (1.17%)	^a^	^a^	20 (1.95%)	^a^	^a^	104 (10.15%)

a Individual results not reported due to small number of cases.

Figure 1 presents the complication rates for 90 days after a TKA for each of the previous gastric bypass types compared with a TKA where no previous gastric bypass took place. Note that the HRs for death, implant failure, periprosthetic infection, and readmission are markedly elevated, although the patient had undergone a previous gastric operation.

**Figure 2:**
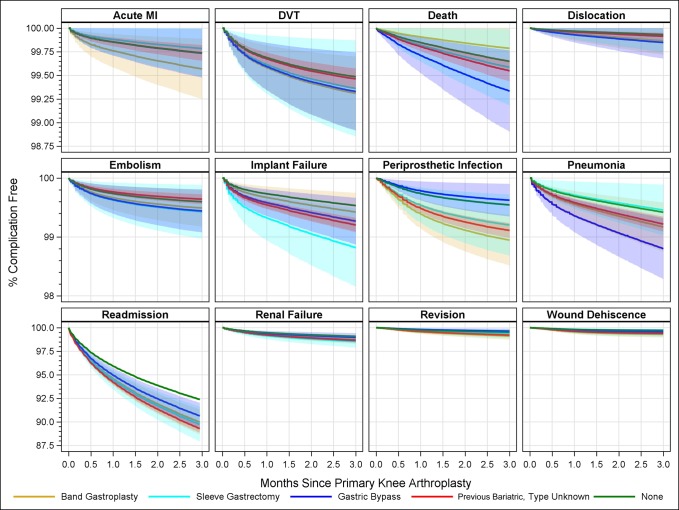
The 90-day complications as a cumulative incidence of percent complication free versus time to 90 days postoperatively.

Figure 2 presents the same 90-day complications as a cumulative incidence. Although revision and wound dehiscence have notable HRs, the baseline values and the increased numbers of complications are both low. What is of concerning is the trajectory and dispersion of the curves for implant failure PJI, pneumonia, and readmission. The readmission rate 90 days postoperatively having had no previous bypass was about 8%; the readmission rate after an unknown type bypass was about 12% or 4% higher.

**Figure 3:**
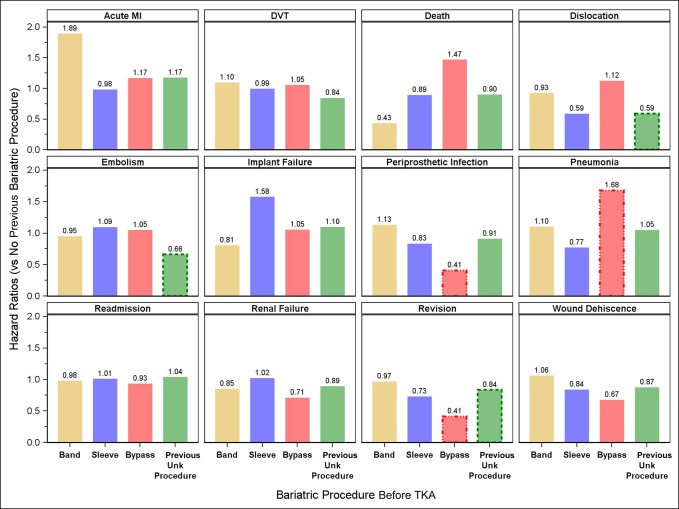
The complication rates, expressed as hazard ratios, for 90 days after a TKA for each of the previous gastric bypass types, compared with patients with morbid obesity (body mass index > 40 kg/m^2^) who have not undergone previous gastric bypass.

Figure 3 presents the complication rates for 90 days after a TKA for each of the previous gastric bypass types compared with a TKA where no previous gastric bypass took place. The comparison group in this figure was limited to the subcohort with a BMI of greater than 40 kg/m^2^. In this more rigid comparison, the readmission rate has not markedly decreased over the patients with morbid obesity who have not undergone bariatric surgery. The exceptions are noted by the HR for pulmonary embolism (previous bypass type unknown), periprosthetic infection for a TKA after a Roux-en-Y bypass (HR 0.41), renal failure (HR 0.71), revision (HR 0.41), and wound dehiscence (HR 0.67) for a TKA after a Roux-en-Y bypass.

## Discussion

Patients with obesity are at increased risk of complications after joint replacement. Patients with obesity of a notable magnitude may be instructed to lose weight before surgery is considered, as an effort in mitigating risk. Given the success bariatric surgery has demonstrated producing weight loss among the super obese, bariatric surgery may indeed be recommended for such patients contemplating joint replacement. The presumed rational for this approach is that curing obesity would subsequently mitigate any attendant risks. This rationale does not account for the possibility that the obesity is a proxy for other underlying diseases and does not normalize the risks associated with these diseases. The literature on this topic is conflicting and has some of the same underpowered limitations encountered with superobesity.

Watts et al,^5^ Inacio et al,^6^ Smith et al,^7^ Parvizi et al,^8^ and Kulkarni et al^9^ done single institution studies involving small numbers of patients for which the conclusions were equivocal. Lee et al^10^ done a Medicare 5% part B study from 1999 to 2012 involving THA (n = 47,895) and primary TKA (n = 86,609). In this cohort, 0.1% or 1,345 patients had undergone a previous bariatric procedure within 2 years. Their conclusion was “the effect of bariatric surgery before elective THA/TKA remains unclear.” Werner et al^11^ done a PearlDiver data mining study for the period of 2005 to 2011. The study cohort included 78,036 unique individuals of whom 11,294 were morbidly obese and 219 underwent previous bariatric surgery. Their conclusion was that “bariatric surgery before TKA appears to be associated with less risk of postoperative complications.” McLawhorn et al.^11^ done a New York Statewide database study involving THA and TKA done between 1997 and 2011. For TKA, 2,636 bariatric surgery patients were matched to 2,636 morbidly obese patients. For THA, 792 bariatric surgery patients were matched with 792 morbidly obese patients. Risks of in-hospital complications were lower for THA and TKA (odds ratio 0.25, *P* < 0.001 and odds ratio = 0.69, *P* = 0.021, respectively). However, bariatric surgery did not reduce the risk of revision surgery for either THA or TKA. Table 5 presents the above studies and summarizes the conclusions.

**Table 5:** Literature Review

	Single Institution	Cohort Size	TKA/THA	Body Mass Index	Conclusion
Watts et al^5^	Yes	47	THA	49.7	May decrease revision surgery and revision
Inacio et al^6^	Kaiser	69/102	TKA/THA	Not specified	May not provide dramatic improvement
Smith et al^7^meta-analysis (5 studies)		657	TKA/THA	Not specified	Questions previous belief that bariatric surgery may improve the clinical outcomes
Lee et al^10^	Medicare data	86,609/47,895	TKA/THA	Not specified	Previous bariatric at higher risk of infection but not revision
Nickel et al^13^	Medicare data	39,014	TKA/THA	Not specified	BS associated with greater risk of both obese and nonobese
Kulkarni et al^9^	English NHS	53/90	TKA/THA	Superobese	Risk seems to be lower
Parvizi et al^8^	Yes	20	TKA/THA	49	Should be considered for bariatrics
Werner et al^11^	PearlDiver	219	TKA/THA	Morbid	Seems to be associated with less risk
McLawhorn et al^12^	NYS database	2,636	TKA/THA	Morbid	BS did not reduce the risk of revision TKA or THA

This large retrospective study of Medicare patients involves 2.7 million patients over an 11-year period. In this cohort, 25,852 patients underwent a gastric bypass before undergoing a TKA. The study was limited to TKA in an effort to maintain uniformity and to eliminate anthropometric factors which may be unique to a different joint. This study was designed to use the same methodology and data presentation as our previous study on obesity and TKA where previous bariatric surgery did not take place.

The outcome variables were selected to correspond with the same outcomes being tracked by Medicare for the critical 90-day postoperative period. For administrative and regulatory purposes, large categories such as medical, surgical, and financial may be appropriate. For surgical purposes, the specific incidences of prosthetic joint infection, wound dehiscence, dislocation, and implant failure are critical events. From a commonly tracked notable medical complication purpose, death MI, DVT/PE, and acute renal failure are critical variables. Although strokes and respiratory failure do occur in the extreme superobese category, these are less common. From the critical bundling, cost of episode of care, readmission, prosthetic joint infection, and revision rates are pertinent.

The data presented in this study demonstrated four worrisome trends:

(1) From a side-by-side comparison between obese patients without a bariatric procedure and those who underwent bariatric surgery before TKA, it appears that complications from prosthetic failure may actually be higher after bariatric surgery. This finding is not unique to knee replacement; Nickel et al^13^ found that the dislocation rate increases with bariatric surgery done before total hip arthroplasty.
(2) The complications of TKA were elevated in almost every category. The few exceptions such death in the “band” category were confounded by the fact that there were no such events. When reviewing the elevated HRs, these must be taken in context of the actual incidence of the event. For example, the HR of wound dehiscence was near two for each type of bypass; yet, the actual number was small.
(3) If one were to include the error bars in the analysis, the all critical hospital readmission rate was not markedly improved for those who underwent a previous bariatric procedure. Although some of the complications plateaued at 90 days, the curves for readmission continued to increase and were divergent for the more aggressive bypasses. The bariatric literature indicates that the laparoscopic sleeve gastrectomy is reserved for lower BMI patients (e.g., 35.0 to 43.0)^13^ and the Roux-en-Y procedure for the higher BMI patients. Comparing the complications for the morbidly obese without a bariatric procedure and a patient with morbid obesity who underwent an LSG would suggest that the risks from an orthopaedic standpoint after a TKA have not been improved after LSG. A similar comparison between a patient with superobesity who has not undergone a Roux-en-Y bypass and one who has would suggest the same conclusion.^14^ A reasonable conclusion would therefore be that the ultimate risk category is based on the highest BMI achieved lifetime and that the type of bypass matters in expected outcomes.^15^

A limitation of this study is that the outcomes are limited to Medicare patients older than 65 years.

A major limitation of our methods is that the individual patient BMIs could not be measured. It is therefore possible that each and every patient who underwent bariatric surgery nevertheless failed to lose weight and may have indeed gained weight. Thus, our study cannot support the conclusion that weight loss is ineffective at modifying the risk profile. Along those lines, although the risks were higher among the bariatric surgery patients as compared to controls, it is unknown whether the risk would have been higher still had bariatric surgery not been undertaken. All we can say is that the patient was allowed to undergo a TKA in the current preoperative screening environment. A more specific list of limitations is contained in our previous study on superobesity and TKA.^4^

The current state of orthopaedic data bank data does not include specifics regarding medication such as TXA, laboratory data such as hemoglobin or transfusion requirements, nutritional status, the actual BMI at the time of the arthroplasty, radiograph findings, or mode of implant failure. Although these limitations may be notable, these limitations should not preclude presentation of the data that is available and to possibly point out the type of data we could be collecting in the future as centralized data collection becomes more mature.

An additional limitation is the inability to identify coding-related diagnoses. For example, if a patient has chronic but compensated borderline kidney disease and is dehydrated waiting for surgery, the serum creatinine may transiently cross into an abnormal zone and be coded as acute renal failure. This claims-based study would be unable to determine that this coding change was not a serious clinical concern.

## Conclusions

(1) Submitting to a bariatric procedure does not normalize complications after a subsequent TKA.
(2) Some of the orthopaedic surgical risks and medical complications are improved when only the patients with a BMI of over 40 kg/m^2^ were considered. The critical hospital readmission rate however remained elevated at near 10%.
(3) The type of bariatric procedure affects the complication rate; in general, higher complications occurred with the more extensive bypass surgery.
(4) We were unable to establish that the higher risks persist due to persistent or recurrently elevated BMI.
(5) In addition to BMI, other factors may be relevant such as lean body mass, body fat distribution, aerobic capacity, medical comorbidities, and nutritional deficiencies.
(6) We suggest that the costs attributed to these added complications not be attributed to deficiencies in care.
